# The Influence of TDP1 Inhibitor Usnic Acid Derivative OL9-116 on the Effects of Topotecan in Human Cells

**DOI:** 10.3390/cimb48040428

**Published:** 2026-04-21

**Authors:** Tatyana E. Kornienko, Arina A. Chepanova, Maria V. Kolobenko, Irina A. Chernyshova, Alexandra L. Zakharenko, Artur S. Venzel, Nadezhda S. Dyrkheeva, Andrey V. Markov, Rashid O. Anarbaev, Konstantin N. Naumenko, Olga A. Luzina, Nariman F. Salakhutdinov, Vladimir A. Ivanisenko, Olga I. Lavrik

**Affiliations:** 1 Institute of Chemical Biology and Fundamental Medicine, Siberian Branch of the Russian Academy of Sciences, 630090 Novosibirsk, Russia; t.kornienko1995@gmail.com (T.E.K.); andmrkv@gmail.com (A.V.M.);; 2 Federal Research Centre Institute of Cytology and Genetics, Siberian Branch of the Russian Academy of Sciences, 630090 Novosibirsk, Russia; 3 Department of Natural Sciences, Novosibirsk State University, Pirogov St., 2, 630090 Novosibirsk, Russia; 4 N. N. Vorozhtsov Novosibirsk Institute of Organic Chemistry of SB RAS, Lavrent’ev ave., 9, 630090 Novosibirsk, Russia; luzina@nioch.nsc.ru (O.A.L.);; 5 The Institute of Biology and Biotechnology, Altai State University, Pr. Lenina 61, 656049 Barnaul, Russia

**Keywords:** DNA repair, Tyrosyl-DNA phosphodiesterase 1 inhibitor, topotecan, TDP1 knockout, usnic acid derivative

## Abstract

Tyrosyl-DNA phosphodiesterase 1 (TDP1) is a key enzyme for the repair of stalled topoi-somerase 1 (TOP1)-DNA complexes. We have previously developed a TDP1 inhibitor, compound OL9-116, which is capable of enhancing the action of the anticancer drug topotecan (TPC), a TOP1 poison, in vitro and in vivo. In this study, the inhibition mode of OL9-116 (uncompetitive) was investigated. We have shown that N-terminal domain of TDP1, which is important for the cell function of TDP1 but is not involved in catalysis directly, reduced the inhibitory potency of OL9-116 probably by influencing the conformation of the enzyme. OL9-116 did not reduce cell viability and did not affect mitochondrial membrane potential. OL9-116 enhanced the cytotoxic/antiproliferative effect of TPC on the panel of tumor cells. This effect was not observed on nontumor cells or TDP1-deficient cells. OL9-116 and TPC had different effects on *TDP1* and *TOP1* gene expression detected by PCR depending on the cell type and the presence of functional TDP1. The direct relation between the effects of the compounds on the gene expression and cell survival was not found. The obtained data indicated a synergistic effect of OL9-116 and TPC, which appeared to be mediated by TDP1 inhibition rather than by an effect on *TDP1* gene expression.

## 1. Introduction

Malignant neoplasms remain a major global health challenge and represent one of the leading causes of mortality worldwide. Despite significant advances in cancer treatment achieved to date through immunotherapy, stem cell transplantation, nanoparticles, radiofrequency ablation, hormone therapy, and other modern modalities, chemotherapy remains relevance. For decades, chemotherapy has been a conventional mainstay of cancer treatment alongside surgery and radiotherapy, which together have contributed to a reduction in cancer incidence and mortality rates [1,2]. However, treatment with highly cytotoxic chemotherapeutic agents is associated with a range of complications and adverse effects. These can range from minor and easily manageable to severe, irreversible conditions that significantly impair patients’ quality of life [3]. Further substantial challenges of chemotherapy include the development of drug resistance in tumor cells and the limited selectivity of the administered drugs.

One of the primary mechanisms underlying the development of tumor cell drug resistance is the activity of DNA repair systems. Chemotherapeutic agents act by damaging the DNA structure of tumor cells, which triggers either its repair or the initiation of cell death processes [4]. For instance, topotecan (TPC), a camptothecin (CPT) derivative, is a clinically used anticancer drug that inhibits the enzyme topoisomerase I (TOP1). TOP1 is an enzyme that alters DNA topology during transcription, replication, DNA repair, and chromatin remodeling [5,6]. TOP1 introduces a single-strand break at the 3′ end of the DNA, allowing the 5′ end to rotate around the intact strand, thereby relaxing DNA supercoiling. In the process, the enzyme forms a covalent bond between tyrosine-723 and the 3′ end of the DNA [7]. TPC stabilizes this covalent TOP1-DNA complex (TOP1cc), converting it into a barrier for replication forks, which leads to the accumulation of DNA damage and subsequent tumor cell death [8,9,10].

However, the efficacy of TPC is limited by the intrinsic resistance of tumor cells, which is mediated by DNA repair pathways [11,12,13]. One of these pathways is associated with the enzyme tyrosyl-DNA phosphodiesterase 1 (TDP1). TDP1 releases DNA from TOP1cc by catalyzing the hydrolysis of the phosphodiester bond between the catalytic tyrosine residue of TOP1 and the DNA phosphate group; this enzyme possesses broad substrate specificity, enabling the removal of various blocking lesions from the 3′ end of DNA [14,15,16,17].

The human TDP1 protein consists of a C-terminal catalytic domain, responsible for the enzyme’s phosphodiesterase activity, and an N-terminal domain (residues 1-148). The functional and structural characteristics of the N-terminal domain remain poorly understood: its three-dimensional structure has not been determined, and its amino acid sequence is less conserved compared to other regions of the enzyme. It is known that this domain has disordered structure. While the N-terminal domain does not participate in catalysis in vitro, it is required for proper TDP1 function in the cells [15,18].

Data suggest that elevated TDP1 expression can reduce the cytotoxicity of camptothecin-based drugs, including TPC, and can also influence cellular sensitivity to these agents [19,20,21]. Furthermore, in the study [22] it was demonstrated that TDP1 deficiency enhances sensitivity to alkylating agents, underscoring TDP1’s role as a limiting factor of therapeutic efficacy. In general, these data indicate that TDP1 represents a promising target for the development of novel combination treatment regimens in oncology, which could enhance tumor cell sensitivity to primary chemotherapeutic agents.

**Figure 1:**
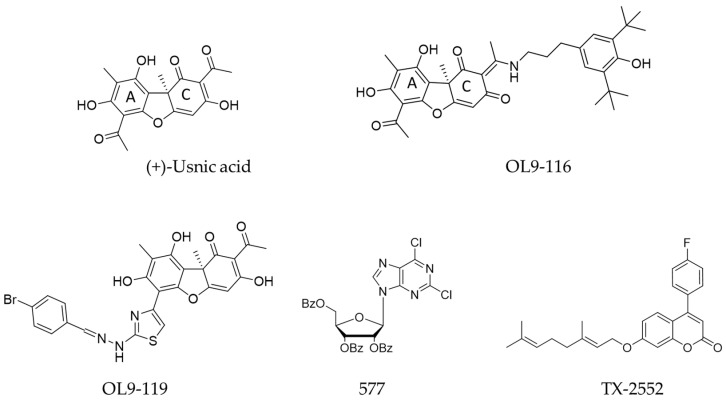
Chemical structures of (+)-usnic acid and Tyrosyl-DNA phosphodiesterase 1 (TDP1) inhibitors which enhanced the effects of topotecan in vivo: enamine (OL9-116) and hydrazonothiazole (OL9-119) derivatives of usnic acid, nucleoside derivative (577) and coumarin derivative (TX-2552). The letters “A” and “C” represent the rings in the usnic acid molecule. More details about these compounds can be found in review [27].

A considerable number of TDP1 inhibitors have been described in the literature. These compounds belong to diverse chemical classes, with the majority representing derivatives of natural bioactive molecules [23,24,25,26,27,28,29,30,31]. We previously identified a compound with laboratory code OL9-116 (Figure 1) as a potent TDP1 inhibitor. This compound is the enamine derivative of usnic acid. It exhibits no detectable toxicity in cultured cell lines or laboratory animals and enhances the antitumor efficacy of a TOP1 poison both in vitro and in vivo [32,33]. In a subsequent study [34], we demonstrated that OL9-116 enhances the effect of TPC in a mouse model of Lewis lung carcinoma (LLC), significantly reducing both tumor volume and the number of metastatic nodules. A similar sensitizing effect was observed in the Krebs-2 ascites carcinoma model, where OL9-116 treatment led to a marked decrease in ascites volume and in the number of viable tumor cells within the ascitic fluid.

The most pronounced synergistic effect (10.5-fold enhancement) was observed for OL9-116 in combination with camptothecin in MTT-assay on MCF-7 cells [33]. Furthermore, studies on two mouse models of transplanted tumors showed that the intragastric administration of OL9-116 enhances the antitumor and antimetastatic activity of TPC [32,34]. We have investigated the pharmacokinetics of the OL9-116 compound in the blood, organs, and tumor nodules of mice [35]. The obtained data allowed us to determine the time to reach the maximum concentration of the substance in the blood, reduce the administered dose of OL9-116, and achieve the best therapeutic outcome in in vivo experiments.

The aim of this work was to investigate the TDP1 inhibitor OL9-116 to determine its sensitizing potential in combination with topotecan. In the present study, we found that OL9-116 compound is less effective in the absence of the TDP1 N-terminal domain (the half-maximal inhibitory concentration (IC_50_) is 2.5 times higher than for the whole form of the enzyme). We also determined the inhibition type and performed molecular modeling to elucidate the mechanism of the inhibitor’s interaction with the enzyme. Furthermore, the contribution of the direct interaction between the inhibitor and TDP1 to the observed synergistic effect required clarification. Within the framework of the hypothesis considering TDP1 as the target for OL9-116, this study investigated its ability to sensitize cells to the cytotoxic/antiproliferative and DNA-damaging effects of TPC. Comparative analysis revealed that the sensitizing effect occurs exclusively in wild-type A549 human lung carcinoma cells and is entirely absent in the TDP1-knockout line. The lack of a sensitizing effect of the TDP1 inhibitor was also observed in the model non-tumorigenic HEK293A cell line, irrespective of the TDP1 status. To determine the mechanism underlying OL9-116-induced sensitization of tumor cells to TPC, we analyzed changes in the expression levels of the *TOP1* and *TDP1* genes in A549 and HEK293A cells. The obtained data support the conclusion that this effect is a direct consequence of TDP1 inhibition rather than modulation of TDP1 or TOP1 expression.

## 2. Materials and Methods

### 2.1. Purification of Recombinant Human TDP1-Δ148 Expressed in Escherichia coli

The recombinant proteins TDP1 and the mutant TDP1 with a truncated N-terminal domain (Δ148TDP1) were isolated and purified according to [36]. *E. coli* Rosetta pLysS cells were used for the expression of the proteins. Plasmids developed in the Laboratory of Bioorganic Chemistry of Enzymes of the Institute of Chemical Biology and Fundamental Medicine of the Siberian Branch of the Russian Academy of Sciences were used: pQE30-Δ148TDP1 plasmid (for Δ148TDP1) and pLate31-TDP1 (for TDP1 wild type).

### 2.2. Evaluation of TDP1 and Δ148TDP1 Enzyme Activity by Real-Time Fluorimetry

The enzymatic activity of TDP1 and Δ148TDP1 was assessed as previously described [37]. Briefly, purified TDP1 in a final concentration of 1.5 nM was added to the reaction mixture (200 µL) containing 50 nM oligonucleotide in buffer (50 mM Tris-HCl, pH 8.0, 50 mM NaCl, and 7 mM β-mercaptoethanol). Fluorescence measurements were performed using a POLARstar OPTIMA fluorimeter (BMG LABTECH, GmbH, Ortenberg, Germany) every 1 min for 7 min (linear portion of the enzymatic reaction rate curve). The inhibitory effect of the tested compounds was evaluated by determining the IC_50_ value (the inhibitor concentration required for 50% enzyme inhibition), which was measured in three independent experiments. Data were processed using MARS Data Analysis 2.0 software (BMG LABTECH).

The assay employs a fluorescent oligonucleotide substrate that mimics damaged DNA: 5′-(5,6 FAM-aac gtc agg gtc ttc c-BHQ1)-3′, where FAM represents the fluorophore (fluorescein amidite) and BHQ1 denotes the quencher (Black Hole Quencher 1). The fluorescence intensity is proportional to the amount of cleaved quencher, reflecting the reaction rate and enabling real-time activity monitoring. This approach allows real-time determination of TDP1 activity, and experimental results were used to calculate the initial reaction rates at different inhibitor concentrations.

### 2.3. Determination of Kinetic Parameters and Inhibition Mode

To determine the inhibition mode of the compounds, TDP1 activity was investigated using a fluorimetric assay at varying concentrations of the substrate, the same oligonucleotide as in Section 2.2 (56 nM, 96 nM, 163 nM, 277 nM, 470 nM, and 800 nM) and in the presence of different inhibitor concentrations (specific for each compound). The obtained data were processed using OriginPro v8.6.0 software (OriginLab Corporation, Northampton, MA, USA). The apparent K_M_ and V_max_ parameters were plotted against inhibitor concentration using Equation (1) to characterize the inhibition type.

(1) $$y=\frac{V_{max}*X}{\left(K_{M}+X\right)}$$

where V_max_ is the maximum rate of an enzymatic reaction, and K_M_ is apparent Michaelis constant.

### 2.4. Determination of Competitive Binding Between the TDP1 Inhibitor and the Oligonucleotide for the Enzyme’s Active Site

Fluorescence anisotropy was determined by measuring the fluorescence intensities parallel and perpendicular to the plane of polarized excitation light. Anisotropy was calculated using Equation (2):

(2) $$r=\frac{\left(I^{∥}-I^{⊥}\right)}{\left(I^{∥}+2I^{⊥}\right)}$$

where r is the fluorescence anisotropy, I^∥^ is the fluorescence intensity parallel to the plane of polarized excitation light, and I^⊥^ is the fluorescence intensity perpendicular to the plane of polarized excitation light.

Fluorescence measurements were performed using a CLARIOstar fluorimeter (BMG LABTECH, GmbH, Ortenberg, Germany). The instrument was calibrated using a fluorescein solution at a concentration 3 nM. The 200 μL reaction mixtures contained buffer (50 mM Tris-HCl, pH 8.0; 50 mM NaCl; 7 mM β-mercaptoethanol), 5 nM oligonucleotide 5′-(5,6 FAM-aac gtc agg gtc ttc c)-3′, and varying concentrations of the inhibitors. The reaction was initiated by adding TDP1 enzyme to a final concentration of 50 nM. Data were processed using MARS Data Analysis 2.0 software (BMG LABTECH).

### 2.5. Analysis of Inhibitor Effects on TDP1-Oligonucleotide Complex Formation

The experiments were performed using an electrophoretic mobility shift assay (EMSA) in a 5% polyacrylamide gel with 0.1% bisacrylamide in 0.3× TBE buffer. Electrophoresis was carried out in vertical plates in 0.3× TBE buffer at a constant current of 4 mA for 40 min.

The reaction mixtures (10 μL) contained buffer (50 mM Tris-HCl, pH 8.0; 50 mM NaCl; 7 mM β-mercaptoethanol), 0.1 μM 5′-5,6 FAM oligonucleotide, 0.5 μM TDP1, and inhibitors at various concentrations. The reaction was initiated by adding TDP1 and incubated for 10 min at 4 °C. Then, 2 μL of Ficoll (Sigma, St. Louis, MO, USA) was added to increase sample viscosity. Fluorescence of the products was visualized by gel scanning on a Typhoon FLA 9500 (GE HealthCare Technologies, Inc., Chicago, IL, USA). Quantitative analysis of band intensity was performed using the QuantityOne v4.6.7 software (Bio-Rad Laboratories, Hercules, CA, USA).

### 2.6. Structural Modeling of TDP1 Interaction with Inhibitor

Two catalytically relevant TDP1 forms were constructed for molecular docking studies: a pre-catalytic TDP1–TOP1cc complex and a phosphohistidine covalent intermediate. The pre-catalytic complex, representing the enzyme-substrate state prior to catalysis, was modeled using the structure of TDP1 bound to a tyrosyl-DNA adduct (PDB ID 1RFF), which contains the peptide KLNYYD (from Topo1) covalently linked to an AGTT tetranucleotide via Tyr723. Resulting structure mimics the physiological TOP1 cleavage complex (TOP1cc) substrate. The phosphohistidine covalent intermediate, representing the catalytic state with phosphohistidine, was derived from the same 1RFF structure. Missing loops in both structures were reconstructed using AlphaFold2 via ColabFold [38,39] in single-sequence mode (without MSA) with 1RFF as a structural template to preserve native conformations while accurately predicting missing regions.

To generate the phosphohistidine covalent intermediate, the vanadate in 1RFF was converted to phosphate in PyMOL v3.1 [40], and a covalent bond was formed between the catalytic His263 (NE2 atom) and the oligonucleotide phosphate (P atom). The Tyr723-containing peptide was removed to reflect the post-cleavage state. For the pre-catalytic TDP1–TOP1cc complex, the native Tyr723-phosphate linkage from 1RFF was retained. Both complexes were refined using PyRosetta 4 [41] with the ref2015 energy function [42]. Covalent bonds were explicitly defined in the topology, followed by energy minimization to optimize side-chain conformations. Local structural relaxation was then performed using the FastRelax protocol on residues within 8 Å of the oligonucleotide, while maintaining the remaining protein backbone fixed. The inhibitor OL9-116 structure was prepared using the RDKit package (https://www.rdkit.org).

Molecular docking of OL9-116 to both TDP1 forms was performed using GNINA v1.3 [43] with the AutoDock4 scoring function [44]. Docking poses were rescored using GNINA’s dense convolutional neural network model, and the binding modes with the highest CNNScore were selected for further refinement. For each protein-ligand complex, 100 independent FastRelax relaxations were carried out. Binding affinities for each relaxed pose were computed as ΔG = ΔGcomplex − (ΔGprotein + ΔGligand), with all energy terms evaluated using the ref2015 scoring function in Rosetta Energy Units (REU). The docking protocol was validated by re-docking co-crystallized ligands into 14 TDP1 crystal structures, yielding a median RMSD of 0.23 Å (Table S1, Figure S3), with the AD4 scoring function showing the strongest correlation with experimental binding affinities (r = −0.79) (Table S2); full details are provided in the Supplementary Materials.

### 2.7. Molecular Dynamics Simulations

To assess the stability of the predicted docking poses, molecular dynamics (MD) simulations were performed for the both catalytic states of TDP1 in complex with OL9-116 and the oligonucleotide cofactor: (1) the pre-catalytic TDP1–TOP1cc complex (DNA covalently linked to Tyr723 of the TOP1-derived peptide) and (2) the phosphohistidine covalent intermediate (DNA covalently bonded to His263). The inhibitor was treated as a non-covalent binder. The AMBER ff14SB force field was used for the protein and peptide, GAFF 2.11 with AM1-BCC charges for the small molecules and DNA, and TIP3P for water. Each system was solvated in a cubic water box with 0.15 M NaCl. After energy minimization and stepwise equilibration, production runs of 100 ns were carried out at 300 K and 1 bar. Three independent replicas were performed for each system. All simulations were carried out in OpenMM 8.1 [45]. Trajectory analysis (RMSD, hydrogen bond occupancy) was performed using MDTraj v1.9.9 [46]. Detailed molecular dynamics protocol is available in Supplementary Materials.

### 2.8. Cell Lines and Culture Conditions

The HCT-116 (human colorectal carcinoma) and MRC-5 (human lung fibroblast) cell lines were provided by the Cell Culture Collection of the State Research Center of Virology and Biotechnology “Vector”, Novosibirsk, Russia. The A549 (human basal alveolar epithelial adenocarcinoma cells), HeLa (human cervical carcinoma), MCF-7 (human invasive ductal breast adenocarcinoma) and T98G (human glioblastoma) cell lines were obtained from the Russian Collection of Cell Cultures (RCCC) at the Institute of Cytology, Russian Academy of Sciences (St. Petersburg, Russia). HEK293A cells (human primary embryonic kidney cells) were purchased from ThermoFisher Scientific (Waltham, MA, USA), cat. No R70507.

All cell lines used in this study were cultured in DMEM-F12 medium (ThermoFisher Scientific, Waltham, MA, USA) supplemented with 100 U/mL penicillin-streptomycin (ThermoFisher Scientific, Waltham, MA, USA), 1× L-alanyl-L-glutamine (GlutaMAX, Gibco, Waltham, MA, USA), and 10% fetal bovine serum (FBS; ThermoFisher Scientific, Waltham, MA, USA) at 37 °C in a humidified atmosphere with 5% CO_2_. The A549 and HEK293A TDP1-knockout clones were generated at the Laboratory of Biochemical Pharmacology, Institute of Chemical Biology and Fundamental Medicine, Siberian Branch of the Russian Academy of Sciences [47,48].

### 2.9. MTT Assay

The cytotoxicity of OL9-116 was evaluated in cell lines using a standard MTT assay [49]. Cells were seeded in plates at a density of 5000 cells per well in DMEM-F12 medium supplemented with 50 U/mL penicillin, 50 µg/mL streptomycin (Thermo Fisher Scientific, USA), and 10% fetal bovine serum (Biolot, Saint Petersburg, Russia), maintained in a 5% CO_2_ atmosphere. Test compounds were added the following day (1:100 reagent-to-total culture medium volume, resulting in a final DMSO (Sigma, St. Louis, MO, USA) concentration of 1%), and the cultures were incubated for 72 h. Control cells were grown in the presence of 1% DMSO. Cytotoxicity measurements were performed in triplicate. Optical density measurements were performed using a CLARIOstar fluorimeter (BMG LABTECH, GmbH, Ortenberg, Germany). Data were processed using MARS Data Analysis 2.0 software (BMG LABTECH). Semi-toxic concentrations of compounds (parameter CC_50_) were calculated using the QuantityOne 4.6.7 software (Bio-Rad Laboratories, Hercules, CA, USA).

### 2.10. Analysis of Mitochondrial Membrane Potential

A549 WT cells were seeded in a 12-well plate and cultured overnight at 37 °C and 5% CO_2_. The cells were then treated with OL9-116 at 5 µM for 24 h. Then the cells were harvested by trypsinization, resuspended in JC1 (LumiTracker^®^ Mito JC-1, Lumiprobe Corporation, Westminster, MD, USA)-containing PBS (5 µg/mL; 10^6^ cell/well), and incubated in a CO_2_ incubator for 30 min. The cells were then washed with PBS and analyzed by flow cytometry, using a NovoCyte Flow Cytometer (ACEA Biosciences Inc., San Diego, CA, USA) and the following characteristics: J-monomers (low potential) on the FITC channel (excitation/emission = 495/519 nm) and J-aggregates (high potential) on the channel PE-Texas Red (excitation/emission = 566/616 nm). For each sample, 10,000 events were collected.

### 2.11. Alkaline Comet Assay

The alkaline comet assay was performed as previously described [50]. Briefly, cells were seeded in 24-well plates at a concentration of 0.05 million/mL. The following day, the cells were treated with the test compounds and incubated for 2 h. The suspension was mixed with 1% molten low-melting-point agarose (CertifiedTM LMAgarose; BIO-RAD, Singapore) and transferred onto glass slides pre-coated with 1% standard agarose (Agarose; Helicon, Moscow, Russia), then allowed to solidify at 4 °C.

The slides were incubated in a lysis solution (2.5 M NaCl, 100 mM EDTA, 10 mM Tris-base, 1% Triton, 5% DMSO, pH 10.0) for 1 h and in an electrophoresis buffer (300 mM NaOH, 1 mM EDTA, pH > 13) for 45 min at 4 °C. Electrophoresis was conducted at 20 V and 450 mA for 10 min on ice. The slides were rinsed with cold water and stained with SYBR Green I (Thermo Fisher Scientific, USA).

Images were acquired using a CELENA S digital microscope (Logos Bio-systems, Inc., Gunpo-si, Gyeonggi-do, Republic of Korea) and analyzed using comet assay analysis software version 1.0 (Trevigen, Inc., Gaithersburg, MD, USA). A minimum of 500 cells per sample were analyzed. DNA damage was assessed as the median % Tail DNA = 100 × (tail fluorescence/total comet fluorescence).

### 2.12. Quantitative RT-PCR

A549 wild type (WT) and HEK293A WT cell lines, along with TDP1-knockout cells, were seeded in 6-well plates at a density of 1–2 × 10^6^ cells per well and incubated for 20–21 h. Two parallel replicates were used for each treatment condition. After 20–21 h of incubation, the cells were subjected to one of the following treatments for 5 h: 1 µM TPC + 1% DMSO, 10 µM compound OL9-116, or their combination.

RNA was isolated using TRIzol (Thermo Fisher Scientific, Waltham, MA, USA) according to the manufacturer’s instructions, resuspended in water, and quantified using a Nanodrop 1000 spectrophotometer (Thermo Fisher Scientific, Waltham, MA, USA). Prior to the reverse transcription reaction, the isolated cellular RNA was treated with DNase I (New England Biolabs, Ipswich, MA. USA) as per the manufacturer’s protocol to remove DNA contaminants, followed by enzyme inactivation with 5 mM EDTA; the RNA samples were stored at −70 °C until further use.

Real-time qRT-PCR was performed as described previously [48]. RT-qPCR was performed using BioMaster RT-PCR SYBR Blue reagent (Biolabmix, Novosibirsk, Russia). The RT-qPCR kit contains buffer with all necessary components (except RNA template and primers), BioMaster mix, and DEPC-treated water for one-step reverse transcription and real-time PCR with fluorescent probes. BioMaster mix includes M-MuLV RH reverse transcriptase (RNA/DNA-dependent, RNase H-deficient) and hot-start HS-Taq polymerase in an optimized ratio. Each 20 µL reaction contained 10 ng RNA, primers, and the enzyme mix. Reactions were performed on a LightCycler 96 instrument (Roche, Basel, Switzerland) with the following protocol: reverse transcription at 45 °C for 1800 s; preincubation at 95 °C for 300 s; 36 cycles of 95 °C for 10 s, 60 °C for 10 s, and 72 °C for 10 s; fluorescence detection at 84 °C for 5 s. Primers for reference and target genes were designed based on NCBI sequences. Experiments were performed in triplicate, using GAPDH and B2M as reference genes. Amplification efficiency, relative expression levels (via the ΔΔCt method), and standard errors were determined using LightCycler 96 software version 1.1.0.1320 (Roche, Basel, Switzerland).

PCR Efficiency Calculation:

$$E=10^{-1/slope},$$

*E*—amplification efficiency, *slope*—slope of the calibration curve.

Calculation of ratio using two reference genes:

$$ratio=\frac{\sqrt{E_{R1}^{{Cq}_{R1}}\times E_{R2}^{{Cq}_{R2}}}}{E_{T}^{{Cq}_{T}}},$$

*E*—amplification efficiency, *Cq*—quantification cycle, *R1*—reference gene 1, *R2*—reference gene 2, *T*—target gene.

Error calculation:

$$S=\sqrt{\frac{{\sum \left(X-\bar{X}\right)}^{2}}{n-1}},$$

*n*—sample size, *X*—each sample value, $\bar{X}$—arithmetic mean.

The primer sequences and amplification results are presented in Table 1.

### 2.13. Statistical Analysis

Statistical processing of Comet assay and RT-qPCR data was carried out using one-way ANOVA (STATISTICA software version 12.5, TIBCO Software Inc., Palo Alto, CA, USA). Post hoc assessment was performed using Tukey’s honestly significant difference (HSD) test. Data with *p* < 0.01 were considered statistically significant. Pairwise comparison of cell survival at different concentrations of TPC in the presence and absence of compound OL9-116 was performed using the Mann–Whitney test. Statistical processing of MTT test data was performed using the OriginPro v8.6.0 software (OriginLab Corporation, Northampton, MA, USA).

## 3. Results and Discussion

### 3.1. Investigation of the OL9-116 Inhibition Type

The discovery of enzyme inhibitors among natural and synthetic compounds, coupled with mechanistic studies of their action, represents a cornerstone of modern drug development. The type of inhibition is determined by the location of the inhibitor binding site (at the substrate binding site or at the allosteric site) and on what stage of the catalytic process the inhibitor binds (before or after the substrate). There are four types of inhibition:

Competitive, where a compound binds with free enzyme and competes with a substrate for binding to the active site, which leads to an increase in the apparent Michaelis constant (K_M_) without affecting the enzyme’s maximum velocity (V_max_);

Noncompetitive, where the inhibitor binds both with free enzyme and enzyme-substrate complex to a site other than the active site. This results in an apparent decrease in V_max_ at constant K_M_.

Mixed, involving superposition of the first two.

Uncompetitive, where a compound interacts with the enzyme–substrate complex at a site other than the active site. This results in an apparent decrease in both V_max_ and K_M_. The decrease in K_M_ occurs due to unproductive substrate binding, resulting in a decrease in free enzyme concentration.

More information about the types of inhibition can be found in [51].

Competitive inhibitors bind to the same site as the natural substrate and can bind to all enzymes within a family that share a similar active site structure, potentially leading to side effects. This type of inhibitors may also exhibit withdrawal syndromes, as observed with hypolipidemic statin drugs [52] and antiandrogens [53]. On the other hand, the required doses of competitive inhibitors are typically lower than those of inhibitors with other mechanisms of action, thereby reducing overall toxicity. Uncompetitive inhibition involves the binding of the inhibitor exclusively to the enzyme-substrate complex. This mechanism is highly specific and precludes interaction with other enzymes possessing structurally similar active sites, thereby reducing the probability of side effects. We investigated the effect of OL9-116 on the kinetics of the TDP1-catalyzed reaction by varying the concentrations of both the substrate and the inhibitor to determine the inhibition type. An oligonucleotide biosensor previously developed by our group [37] was used as the substrate. The oligonucleotide contained a fluorophore (FAM) at the 5′ end and a fluorescence quencher (BHQ1) at the 3′ end. TDP1 cleaves the quencher leading to fluorescence increase thus allowing the real-time measurement of enzyme activity. The experiment yielded reaction rate (V) versus substrate concentration (S) dependencies at five different inhibitor concentrations, thus we determined the corresponding kinetic parameters (Figure S1, Supplementary Materials). The data show that both the apparent Michaelis constant and the maximum reaction velocity decrease with increasing inhibitor concentration, indicative of an uncompetitive inhibition mechanism (Table 2).

### 3.2. Study of Competition Between OL9-116 and DNA for the Active Site of the Enzyme

To further characterize the inhibition type, we employed electrophoretic mobility shift assay (EMSA) and fluorescence anisotropy to assess the effect of OL9-116 on TDP1 binding with oligonucleotide substrate.

**Figure 2:**
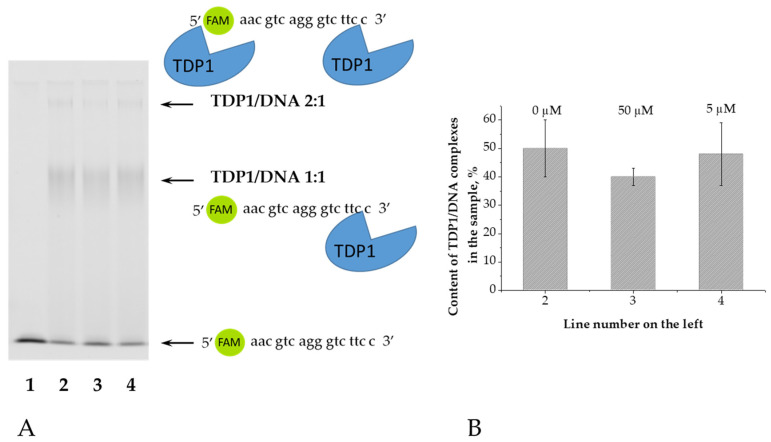
Analysis of the effect of OL9-116 on the stability of TDP1/DNA complexes. (**A**) Electropherogram of TDP1/DNA complex formation products in the presence of OL9-116: 1—DNA only at a concentration of 0.1 μM; 2—mixture of TDP1 0.5 μM and DNA 0.1 μM; 3—TDP1/DNA mixture in the presence of 50 μM OL9-116; 4—TDP1/DNA mixture in the presence of 5 μM OL9-116. Arrows indicate TDP1/DNA complexes. (**B**)—Histogram of the total content of TDP1/DNA complexes from Figure 2A. Above the bars, the concentration of OL9-116 in the samples is indicated.

An oligonucleotide labeled with a fluorophore at the 5′-end of the DNA was used as a substrate for TDP1. Since the cleavage of a natural nucleotide from the 3′ end of DNA by TDP1 occurs much more slowly than that of bulky adducts [54], this substrate can be considered non-cleavable under our experimental conditions. Electrophoresis was performed under native conditions in a 5% polyacrylamide gel. Electrophoretic separation of samples containing the enzyme and the oligonucleotide revealed the formation of two products with different mobility (Figure 2A). TDP1 is capable of processing phosphotyrosyl peptides linked to the 5′ end of DNA [55]. We propose that the enzyme initially binds to the 3′ end of DNA (TDP1/DNA 1:1), and subsequently, when present in excess, binds to the 5′ end (TDP1/DNA 2:1). The total amount of these complexes was calculated relative to the fluorescent signal in each lane (Figure 2B). The amount of TDP1/DNA complexes does not decrease even after adding a high concentration of OL9-116 (50 μM), indicating no competition between DNA and the TDP1 inhibitor.

Fluorescence anisotropy is determined by the difference in light intensity emitted by a fluorophore along different polarization axes. Upon irradiation of the fluorophore with plane-polarized light, molecules oriented in a specific manner relative to the polarization axis preferentially transition to the excited state. If the fluorophore remains immobile, the emitted light will be polarized in the same plane as the absorbed light, resulting in maximal anisotropy. When mobile, the fluorophore changes its position during the excited state lifetime, and the emitted light becomes polarized in other planes. As the fluorophore’s mobility increases, the anisotropy level decreases, since a greater number of molecules emit partially depolarized light [56]. The fluorescence anisotropy of reaction mixtures containing free DNA is minimal, as the fluorophore is bound only to the DNA molecule and rotates relatively rapidly. Addition of TDP1 leads to an increase in anisotropy level, reflecting the formation of an enzyme-DNA complex and, consequently, reduced fluorophore mobility. If an inhibitor capable of displacing the substrate from the enzyme’s active site is added to the reaction mixture, this will result in decreased anisotropy.

We used the same oligonucleotide with a fluorophore at the 5′-end as for EMSA. Upon addition of the compound OL9-116 to the mixture of enzyme and DNA, no changes in anisotropy were observed within the entire range of inhibitor concentrations (Table 3), that also indicates the absence of competition between the inhibitor and DNA for the binding to the enzyme. These results obtained by EMSA and fluorescence anisotropy measurement methods support an uncompetitive mechanism (Section 3.1, Table 2).

### 3.3. Molecular Modeling of TDP1-Inhibitor Interactions

**Figure 3:**
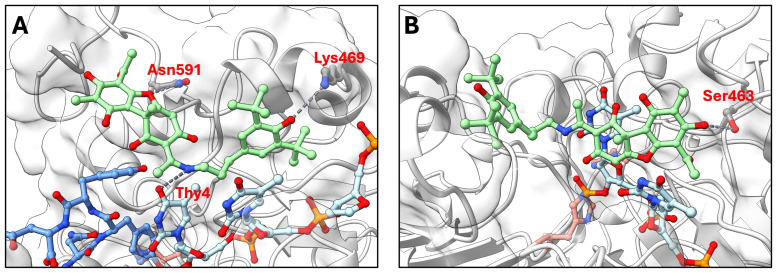
Molecular docking of OL9-116 to TDP1 structures representing two catalytic states. Predicted binding poses of OL9-116 (green sticks) with (**A**) pre-catalytic TDP1–TOP1cc complex and (**B**) phosphohistidine covalent intermediate. Both structures contain an AGTT oligonucleotide (light blue sticks). The catalytic residues His263 and His493 are highlighted in pale red. Potential hydrogen bonds between OL9-116 and TDP1 residues are shown as dark grey dashed lines. In panel (**A**), TDP1 is bound to tyrosyl-DNA adduct representing TOP1cc, comprising a TOP1-derived peptide (KLNYYD, blue) covalently linked to the oligonucleotide via Tyr723 (modeled from PDB ID 1RFF). In panel (**B**), the phosphohistidine intermediate shows His263 covalently bonded to the oligonucleotide phosphate following cleavage of the tyrosyl-DNA bond. Oxygen, nitrogen, and phosphorus atoms are colored red, blue, and orange, respectively.

To gain structural insights into the uncompetitive inhibition mechanism observed experimentally, we modeled the interaction of OL9-116 with two catalytically relevant TDP1 states: (1) the pre-catalytic TDP1 complex with a tyrosyl-DNA adduct representing the physiological TOP1 cleavage complex (TOP1cc) substrate (the peptide KLNYYD from TOP1 covalently linked to an AGTT tetranucleotide via Tyr723), and (2) the phosphohistidine covalent intermediate formed after cleavage of the tyrosyl-DNA bond. Molecular docking using GNINA revealed distinct binding modes for these two states (Figure 3).

In the pre-catalytic TDP1 complex (Figure 3A), molecular docking predicted that OL9-116 binds at the enzyme-substrate interface. The top-ranked GNINA poses were positioned at this interface, where the inhibitor could interact with both the protein and DNA components. The predicted binding mode with the highest docking score shows potential hydrogen bonds with Asn591 and Lys469, as well as interactions with the thymine base of the oligonucleotide, yielding a binding affinity of −27.9 REU (Rosetta Energy Units). This favorable binding affinity suggests that the inhibitor stabilizes the TDP1-substrate complex by engaging both the protein and the DNA substrate simultaneously.

In contrast, in the phosphohistidine covalent intermediate (Figure 3B), the top-ranked predicted pose of OL9-116 has a substantially less favorable binding affinity (−8.92 REU). The predicted interactions involve fewer detectable contacts, with only Ser463 contributing a potential hydrogen bond. This reduction in binding affinity suggests that the covalent intermediate is less compatible with inhibitor binding. Whether OL9-116 additionally delays the ligation step by trapping TDP1 on DNA warrants further investigation.

**Figure 4:**
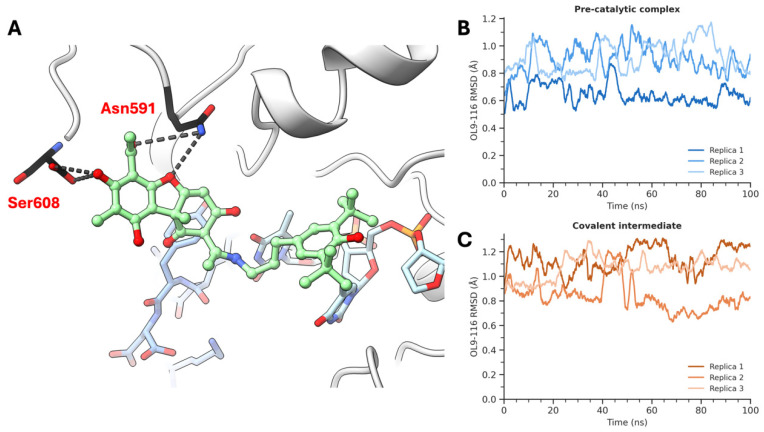
Molecular dynamics simulations of OL9-116 in complex with TDP1. (**A**) Representative snapshot from the pre-catalytic complex (Replica 1, t = 83 ns) showing key hydrogen bond interactions between OL9-116 (green), Ser608 and Asn591 (black). DNA is shown in light blue; TOP1-derived peptide in cornflower blue. H-bonds depicted as black dashed lines. Oxygen, nitrogen, and phosphorus atoms are colored red, blue, and orange, respectively. (**B**) Ligand RMSD time series for OL9-116 in the pre-catalytic TDP1–TOP1cc complex across three independent 100 ns replicas. (**C**) Ligand RMSD time series for OL9-116 in the phosphohistidine covalent intermediate across three independent 100 ns replicas.

To further validate these predictions, molecular dynamics (MD) simulations were performed for both TDP1 catalytic states in complex with OL9-116. Three independent 100 ns replicas were run for each system under NPT conditions. In both cases, OL9-116 remained stably bound throughout the simulations, with mean ligand RMSD values of 0.82 ± 0.15 Å for the pre-catalytic TDP1–TOP1cc complex (Figure 4B) and 1.01 ± 0.16 Å for the phosphohistidine covalent intermediate (Figure 4C), confirming the stability of the predicted binding modes.

Hydrogen bond analysis (Table S3) revealed distinct interactions in each catalytic state. In the phosphohistidine covalent intermediate, the dominant contact was with Ser463—consistent with the docking prediction—along with Tyr204 and minor contributions from Trp590. In the pre-catalytic TDP1–TOP1cc complex, MD simulations refined the docking-predicted binding mode: while the overall binding pose and the Asn591 contact were conserved, the Lys469 hydrogen bond predicted by static docking was not maintained in explicit solvent, and Ser608 emerged as an additional interaction partner (Figure 4A). Overall, the favorable binding energies predicted by PyRosetta, the low ligand RMSD values observed in MD simulations, and the experimental inhibition data are mutually consistent, supporting the proposed binding modes of OL9-116 in both catalytic states of TDP1. The stronger predicted affinity for the substrate-bound state (−27.9 REU) compared to the covalent intermediate (−8.92 REU), together with the ability of OL9-116 to contact both the protein and the DNA substrate, is consistent with the uncompetitive inhibition mechanism observed experimentally.

### 3.4. Investigation of TDP1 and Δ148TDP1 Activity: Determination of IC_50_ Values for Compound OL9-116 Using Real-Time Fluorescence Assay

TDP1 possesses an unstructured N-terminal domain which functions remain incompletely understood. In the study by Interthal et al. [17], it was reported that the activity of the truncated form lacking the first 148 N-terminal amino acid residues (Δ148 TDP1) is practically indistinguishable from that of full-length TDP1 in vitro. Whether the N-terminal domain interacts with TDP1 inhibitors remains unknown. To evaluate the inhibitory properties of the proposed compound, an assay was used based on the TDP1-mediated removal of a fluorescence quencher from the 3′ end of a single-stranded oligonucleotide [37]. Upon quencher removal, fluorescence intensity increases and can be measured in real time. For the investigated compound OL9-116, IC_50_ values 1.4 ± 0.4 μM for the wild-type enzyme and 3.8 ± 0.8 μM for the truncated form Δ148TDP1 were determined. IC_50_ value for Δ148TDP1 is 2.7-fold higher, indicating that the inhibitory potency of the compound depends on the presence of the enzyme’s N-terminal domain, despite its remoteness from the active site. It is possible that the absence of the N-terminal domain leads to a conformational change in the enzyme that is critical for binding of compound OL9-116.

### 3.5. Evaluation of Cytotoxicity and the Ability of OL9-116 to Enhance the Effect of Topotecan on Human Tumor and Non-Tumor Cell Lines

Assessment of the cytotoxicity/antiproliferative effects of compounds on panels of tumor cells represents a fundamental stage in the development of the drugs. It is also important to evaluate these parameters for non-tumor cell lines, as they aid in predicting potential toxicity to healthy tissues.

The cytotoxic/antiproliferative properties of OL9-116 were evaluated using the MTT assay [49]. Given that TDP1 inhibitor-based combination therapies must avoid exacerbating severe treatment-related toxicities, we assessed its safety. OL9-116 exhibited no significant effects on cell viability across multiple human tumor and non-tumor cell lines (HCT-116, A549, MCF-7, T98G, HeLa, MRC-5, HEK293A) at concentrations up to 100 μM, with >80% cell viability maintained under all tested conditions.

We have previously shown that compound OL9-116 (compound 8 in ref. [33]) sensitizes A549 and MCF-7 cells to camptothecin. To investigate the sensitization of the cells by compound OL9-116 to TPC, we evaluated the CC_50_ parameter of TPC both in the absence and presence of OL9-116. It should be noted that HEK293A cells showed high sensitivity to topotecan, which is probably associated with the high level of expression of the SLFN11 protein in these cells [57], which determines the sensitivity of cells to replicative stress [58].

At a concentration of 5 µM, OL9-116 significantly potentiated the effect of TPC in all tested tumor cell lines (Table 4). The most pronounced effect (a 7-fold decrease in CC_50_) was observed in A549 lung cancer cells. It should be noted that TPC is an approved treatment for lung cancer (U.S. Food and Drug Administration. Topotecan Injection: Prescribing Information. Available online: https://www.accessdata.fda.gov/drugsatfda_docs/label/2014/022453s002lbl.pdf; accessed on 20 February 2026). A substantial potentiation (approximately 3-fold) was also evident in HCT-116 colorectal carcinoma, MCF-7 breast cancer, and HeLa cervical carcinoma cells. TPC is used as a first-line therapy for cervical cancer (Ministry of Health of the Russian Federation. Cervical Cancer. Clinical Guidelines. Available online: https://cr.minzdrav.gov.ru/preview-cr/537_3; accessed on 20 February 2026). Notably, the TDP1 inhibitor OL9-116 failed to potentiate TPC-induced cytotoxicity in noncancerous HEK293A and MRC-5 cell lines, indicating tumor-selective chemosensitization. It is noteworthy that HEK293A cells demonstrated considerable sensitivity to TPC (CC_50_ = 16 nM), whereas the CC_50_ values for the other cell lines were in the micromolar range. As we will show below, this phenomenon may be attributed not only to the inhibition of TOP1 by TPC but also to a downregulation of TOP1 expression induced by TPC in these cells.

The observed lack of effect of OL9-116 on TPC cytotoxicity in cells of non-tumor origin (MRC-5 and HEK293A), in contrast to cancer cells, may indicate the compound’s potential selectivity for tumor cells. The obtained data suggest that this selectivity is based on inherent differences between the cell types, particularly in the activity of metabolic pathways and DNA repair mechanisms.

### 3.6. Effect of OL9-116 on the Mitochondrial Membrane Potential

**Figure 5:**
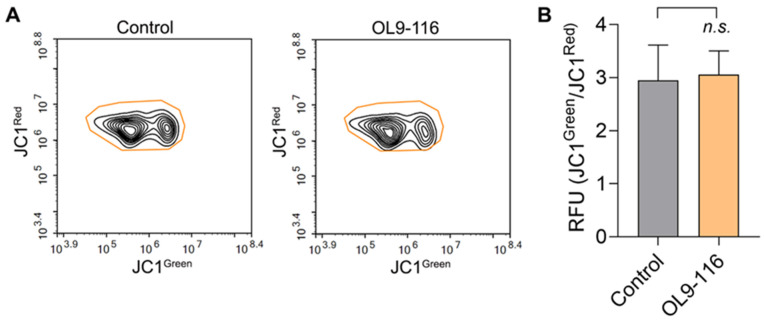
Effect of OL9-116 on mitochondrial membrane potential in A549 cells. (**A**) Cytograms showing the distribution of cells based on red and green fluorescence intensity. (**B**) A bar plot showing the ratio of green to red fluorescence in control and OL9-116-treated cells, indicating no effect of OL9-116 on mitochondrial membrane potential. RFU—relative fluorescence units.

OL9-116 is a derivative of usnic acid, which has been demonstrated to uncouple oxidative phosphorylation, induce adenosine triphosphate (ATP) depletion, decrease glutathione (GSH) levels, and promote oxidative stress, markedly leading to lipid peroxidation and organelle stress [59]. Usnic acid also stimulates mitochondria-derived ROS production via inhibition of complexes I and III of the mitochondrial respiratory chain (MRC) [60] and reduces the mitochondrial membrane potential [61]. In our recent work, we have shown that another usnic acid derivative, OL9-119, which retains the intact C-ring of the parent compound (Figure 1), decreased the mitochondrial membrane potential (the ratio between green and red fluorescence was three times higher than in the control sample) and downregulated genes associated with mitochondrial function [62]. In contrast, in compound OL9-116 the C-ring responsible for mitochondrial toxicity has been modified with an enamine group. This modification resulted in a sharp reduction of the compound’s cytotoxicity compared to both native usnic acid and its derivatives with an intact C-ring [33]. To confirm the absence of mitochondrial toxicity and the enhanced safety profile of OL9-116, we performed a JC1 assay to evaluate its effect on the mitochondrial membrane potential (ΔψM) in A549 cells. The JC1 dye forms red fluorescent J-aggregates in polarized mitochondria, but upon depolarization, it diffuses into the cytoplasm as green fluorescent monomers. As shown in Figure 5, incubation of A549 wild-type cells with 5 µM OL9-116 did not alter the mitochondrial membrane potential, confirming that OL9-116 has no detrimental effect on mitochondria.

### 3.7. Effect of the OL9-116 Compound on Topotecan’s Action in TDP1-Deficient Cell Lines

Substantial evidence indicates that cells deficient in the TDP1 protein (either through knockout or the SCAN1 mutation) exhibit hypersensitivity to camptothecin (CPT) and its analogs, such as topotecan and irinotecan [63,64,65]. The primary underlying cause of this hypersensitivity is the inability of such cells to efficiently repair stabilized TOP1cc, which are the primary targets of these drugs. Numerous experiments, including studies using cellular and animal models, demonstrate that under conditions of impaired TDP1 activity DNA breaks accumulate, consequently enhancing apoptosis [65,66,67]. TDP1 deficiency is a factor that potentiates the cytotoxic effect of TOP1 poisons [68,69,70]. For instance, studies utilizing CRISPR/Cas9 technology to knockout the TDP1 gene in MCF7 (breast cancer), H460 (lung cancer) [71], HEK293A (embryonic kidney) [47], and A549 (lung cancer) [48] cell lines have consistently recorded a substantial decrease in the CC_50_ value for topotecan.

**Figure 6:**
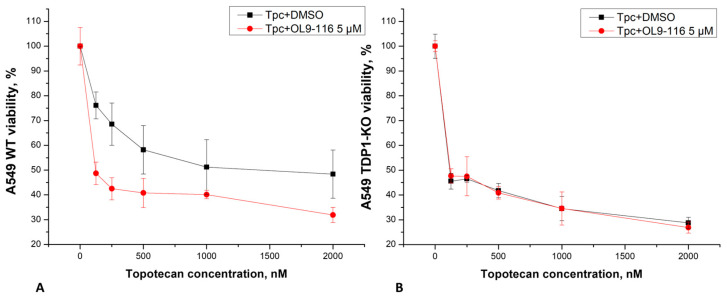
Viability of A549 WT (**A**) and A549 TDP1-KO (**B**) cells, as determined by a standard MTT assay. Combined treatment with TPC and OL9-116 reduced the viability of A549 WT cells, whereas no synergistic effect was observed in A549 TDP1-KO cells.

We investigated whether the compound OL9-116 could enhance the effect of topotecan (TPC) in A549 wild-type (WT) and TDP1-knockout (TDP1-KO) cell lines, the latter being TDP1-deficient cells generated using CRISPR/Cas9 technology. The production of A549 TDP1-KO cells is described in [48], the doubling time of A549 WT and A549 TDP1-KO cells was almost equal and amounted to about 20 h (Figure S4). Analysis of MTT assay data revealed that OL9-116 at a non-toxic concentration of 5 μM potentiated the cytotoxic effect of TPC in wild-type A549 cells by 7-fold (Figure 6). In experiments using the A549 TDP1-KO line, the viability curves following treatment with TPC alone and its combination with OL9-116 overlapped, indicating no synergistic effect. These results suggest the potential of OL9-116 as a TDP1 inhibitor for use in therapy as a TPC sensitizer; however, further studies are required to determine the optimal ratio of components.

The mechanism of topotecan involves its binding to TOP1cc, thereby preventing the restoration of DNA integrity after TOP1 relieves torsional stress in the DNA molecule. This process results in replication fork collapse, double-strand breaks, and subsequent apoptosis. TDP1 serves as a key repair enzyme that counteracts the effects of TPC through the hydrolysis of the phosphodiester bond between the TOP1 tyrosine residue and the 3′ end of DNA within the TOP1cc, facilitating further repair by enzymes such as polynucleotide kinase phosphatase (PNKP) [72]. To evaluate the extent of DNA damage in A549 WT cells and A549 TDP1-KO cells treated with TPC alone or in combination with OL9-116, the alkaline comet assay was employed.

**Figure 7:**
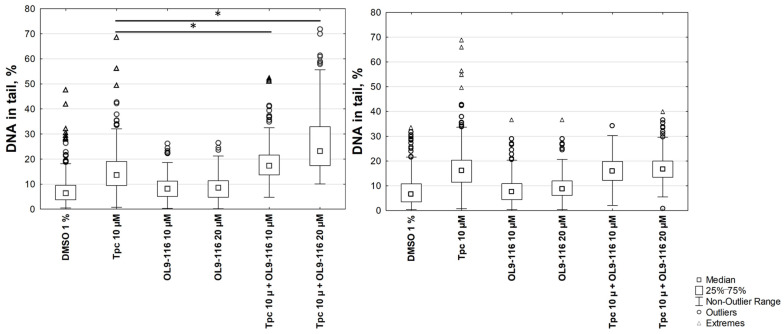
DNA damage levels assessed using the alkaline comet assay. The percentage of DNA in the tail was statistically significantly increased (*p* < 0.01) following combined treatment in A549 WT cells (**A**) compared to treatment with TPC alone. In A549 TDP1-KO cells (**B**), no such effect was observed. * *p* < 0.05.

The results demonstrated that the percentage of DNA in the tail for A549 WT cells following treatment with the compound OL9-116 at concentrations of 10 μM and 20 μM (8.3 ± 3% and 8.4 ± 3%, respectively) did not significantly differ from the 1% DMSO control (6.7 ± 2.9%). When cells were treated with OL9-116 in combination with TPC, a statistically significant (*p* < 0.01, Tukey’s test) increase in DNA damage levels was observed (Figure 7): the percentage of DNA in the tail reached up to 18 ± 5% at 10 μM OL9-116 and up to 27 ± 6% at 20 μM OL9-116, compared to 15 ± 4% with TPC treatment alone. In A549 TDP1-KO cells, TPC treatment elevated the DNA content in the tail to 19 ± 7%, whereas OL9-116 exerted no influence on TPC-induced DNA damage in cells with TDP1 gene knockout (OL9-116 10 μM: 8 ± 3.9%; OL9-116 20 μM: 9.6 ± 3.9%). When TDP1-KO cells were treated with OL9-116 at 10 μM in combination with TPC, the percentage of DNA in the tail was 15.9 ± 4.3%, and at 20 μM OL9-116 it was 17.3 ± 4.3%.

The results of the MTT assay and alkaline comet assay demonstrate that the compound OL9-116 potentiates the cytotoxic effect of TPC in the A549 WT cell line. This synergistic effect is dependent on the presence of TDP1 because no statistically significant enhancement of TPC’s action was observed in the A549 TDP1-KO cell line. The obtained data indicate that the molecular target of the synergy between OL9-116 and TPC is the inhibition of TDP1.

### 3.8. Analysis of the Expression of TDP1 and TOP1 Genes Under the Action of Compound OL9-116 and Its Combination with TPC

The important role of TDP1 and TOP1 enzymes in DNA repair and replication underlie their significant influence on carcinogenesis and tumor cell sensitivity to chemotherapeutic agents. Elevated expression of these genes in tumor tissues may contribute to genomic instability, enhanced DNA repair efficiency, and the development of resistance to TOP1 inhibitors—particularly camptothecin derivatives (topotecan and irinotecan). Bai et al. (2016) reported that hyperexpression of TOP1 and TOP1MT (mitochondrial topoisomerase 1) occurs in 30–70% of epithelial ovarian cancer cases and may hold prognostic value [73]. In olaparib-resistant ovarian cells, TOP1 activity increases while TDP1 activity decreases, thereby enhancing irinotecan sensitivity [74]. Barthelmes et al. (2004) further observed that TDP1 hyperexpression does not affect cellular proliferation but may represent a mechanism of pleiotropic resistance in cancer therapy [20]. Consequently, it was critical to determine whether OL9-116 cellular effects correlate with altered expression of these enzymes.

In this study, we employed quantitative reverse transcription PCR (qRT-PCR) to assess the expression levels of *TDP1* and *TOP1* mRNA in HEK293A, A549 WT, and TDP1-KO cells following treatment with compound OL9-116, TPC, or their combination. The HEK293A TDP1-KO cell line was generated previously [47]. Despite the presence of non-functional mRNA in HEK293A KO cells, the absence of TDP1 protein was demonstrated by Western blot (Figure S2). The obtained data allowed us to evaluate the contribution of these genes to the molecular basis of OL9-116′s ability to sensitize tumor cells to topotecan.

In HEK293A WT and A549 WT cells, topotecan treatment reduced the expression of both *TDP1* and *TOP1* genes by 1.5- to 5-fold (Table 5), and this reduction occurred independently of the presence of OL9-116. It is interesting that, unlike OL9-116, the presence of another TDP1 inhibitor, compound 6d, abrogated the effect of topotecan on wild-type cells [48]. Next, we demonstrated that compound OL9-116 does not affect or has little effect on *TDP1* and *TOP1* gene expression in WT cells.

In knockout cells, the situation is dramatically different: no treatment results in significant changes in the expression of either gene, with the exception of TDP1 expression in A549 cells, which fell 20-fold (Table 5). These data indicate that in HEK293A TDP1-KO cells the compounds and/or TPC-induced TOP1-DNA adducts exert a regulatory influence on *TOP1* and *TDP1* gene expression exclusively in the presence of the TDP1 protein.

Regarding A549 cells, such a strong decrease in expression may indicate the extreme instability of the *TDP1* transcript in this clone, possibly due to degradation (for example, using microRNAs). The same decrease in *TDP1* expression for different types of treatment (TPC, another TDP1 inhibitor, a lipophilic nucleotide derivative, and their combination) was observed in A549 TDP1-KO cells previously [48].

Results obtained by qRT-PCR analysis demonstrate that compound OL9-116 and TPC differentially affect the expression of *TDP1* and *TOP1* genes depending on cell type and the presence of functional TDP1. Overall, OL9-116 has little or no effect on the expression level of these genes in wild-type cells, but its combination with TPC was found to enhance *TDP1* suppression, particularly in HEK293A WT cells. This suggests the existence of cell-type-specific regulatory mechanisms where reduced expression may contribute to increased sensitivity to DNA-damaging agents, although these effects vary and warrant further investigation to understand their clinical implications. The ability of OL9-116 to sensitize cells to topotecan appears to be mediated primarily through TDP1 inhibition rather than through modulation of *TDP1* gene expression.

## 4. Conclusions

In this study, we characterized the inhibition mechanism of OL9-116, a selective inhibitor of the DNA repair enzyme tyrosyl-DNA phosphodiesterase 1 (TDP1) [33]. We have obtained the following new information about the mechanism of action of this compound:

- OL9-116 exhibited uncompetitive inhibition, suggesting it binds exclusively to the TDP1-substrate complex. This mechanism may enhance target specificity and reduce interference with structurally related enzymes, potentially mitigating off-target toxicities.
- Furthermore, we established that the N-terminal domain of TDP1, which is important for the enzyme’s function in the cell but is not involved in catalysis directly, influences the inhibitory potency of OL9-116. It is possible that the absence of the N-terminal domain induces a conformational change in the enzyme that is critical for OL9-116 binding to TDP1-substrate complex.
- We demonstrated that OL9-116 does not affect cellular metabolic activity or mitochondrial membrane potential, indicating its safety profile. This compound enhances the cytotoxic/antiproliferative effect of topotecan on a panel of tumor cell lines, but not on non-tumor cells. The observed lack of effect of OL9-116 on TPC cytotoxicity in non-tumor HEK293A and MRC-5 cells, in contrast to tumor cell lines, may indicate a potential selectivity of this compound for tumor cells.
- To test the hypothesis that OL9-116 acts through a TDP1-dependent mechanism, we assessed its ability to potentiate the cytotoxic and DNA-damaging effects of TPC in A549 WT and A549 TDP1-KO cells. OL9-116 significantly enhanced topotecan-induced cytotoxicity and DNA damage in wild-type cells but showed no potentiation in TDP1-KO cells.
- qRT-PCR data analysis indicates that compound OL9-116 and TPC differentially affect the expression of TDP1 and TOP1 genes, depending on the cell type and the presence of functional TDP1. Compound OL9-116 had little or no effect on the expression of these genes and had no effect on the effect of topotecan in either cell type, regardless of TDP1 status. The only exception was TDP1-deficient A549 cells, where the unproductive TDP1 expression was suppressed under all treatment conditions. The significance of this finding remains unclear and warrants further investigation.

The obtained data indicate a synergistic effect between OL9-116 and TPC, which appears to be mediated by the inhibition of the TDP1 enzyme rather than through an effect on TDP1 gene expression. These results underscore the key role of the TDP1 enzyme as a potential therapeutic target for enhancing the efficacy of TOP1 inhibitor-based therapy.

## Figures and Tables

**Table 1:** Real-time PCR efficiency and primer sequences for human genes.

Genes	Primer Sequence, 5′→ 3′	PCR Efficiency (A549)	PCR Efficiency (HEK293A)
*GAPDH*	AGATCATCAGCAATGCCTCCT	1.86	2.01
	TGGTCATGAGTCCTTCCACG		
*B2M*	CGCTCCGTGGCCTTAGCTGT	1.87	1.95
	AAAGACAAGTCTGAATGCTC		
*TOP1*	CCTCCTGGACTTTTCCGTGG	2.07	2.00
	GGAACCTTGGCATCTTTGCTAC		
*TDP1*	AAGACATCTCTGCTCCCAATG	2.2	2.17
	TTCCCTTTATCCAGCATGTCC		

**Table 2:** Apparent values of maximum reaction velocity (V_max_) and Michaelis constant (K_M_) for compound OL9-116 derived from the velocity versus substrate concentration V(S) plots.

OL9-116 Concentration, µM	Apparent V_max_, c.u.	Apparent K_M_, nM
0	0.42 ± 0.20	40 ± 25
0.3	0.34 ± 0.13	30 ± 17
0.5	0.32 ± 0.15	28 ± 20
0.55	0.19 ± 0.07	11 ± 7
0.6	0.12 ± 0.03	4 ± 3
0.75	0.07 ± 0.02	0.4 ± 0.4

**Table 3:** Fluorescence anisotropy values in the presence of different concentrations of OL9-116.

Concentration of OL9-116, μM	Fluorescence Anisotropy, c.u.
0	80 ± 3
0.55	79 ± 1
1	80 ± 2
1.5	81 ± 3
2.3	79 ± 3

**Table 4:** Semi-toxic concentrations CC_50_ values of topotecan, μM *.

Combination	HCT-116	A549	MCF-7	T98G	HeLa	HEK293A	MRC-5
TPC + DMSO	0.81	1.5	1.4	2.5	9.6	0.016	2.1
TPC + OL9-116	0.32	0.2	0.55	2	3.3	0.017	2.2

* Experiments were performed in at least three replicates, with errors not exceeding 20%.

**Table 5:** Relative expression levels of *TDP1* and *TOP1* genes in HEK293A and A549 cells (wild type and TDP1-KO) treated with TPC, OL9-116 compound and their combination. All data are normalized to the corresponding cells without treatment.

	A549 WT	A549 WT TPC	A549 WT OL9-116	A549 WT TPC + OL9-116
TDP1	100 ± 8	42 ± 8	65 ± 10	35 ± 10
TOP1	100 ± 1	33 ± 3	74 ± 7	64 ± 4
	HEK293A WT	HEK293A WT TPC	HEK293A WT OL9-116	HEK293A WT TPC + OL9-116
TDP1	100 ± 8	67 ± 4	103 ± 7	32 ± 3
TOP1	100 ± 4	18 ± 1	94 ± 2	16.5 ± 0.7
	A549 TDP1-KO	A549 TDP1-KO TPC	A549 TDP1-KO OL9-116	A549 TDP1-KO TPC + OL9-116
TDP1	100 ± 15	2.2 ± 0.08	2.8 ± 1.3	1.9 ± 0.4
TOP1	100 ± 7	91 ± 8	138 ± 5	89 ± 4
	HEK293A TDP1-KO	HEK293A TDP1-KO TPC	HEK293A TDP1-KO OL9-116	HEK293A TDP1-KO TPC + OL9-116
TDP1	100.0 ± 0.3	123 ± 9	110 ± 7	107 ± 9
TOP1	100 ± 5	103 ± 5	134 ± 3	103 ± 4

## Data Availability

The original contributions presented in this study are included in the article/Supplementary Material. Further inquiries can be directed to the corresponding author(s).

## Supplementary Materials

The following supporting information can be downloaded at: https://www.mdpi.com/article/10.3390/cimb48040428/s1. References [43,75,76,77] are cited in the Supplementary Materials.

## Abbreviations

The following abbreviations are used in this manuscript:

CPT	camptothecin
SCAN1	Spinocerebellar Ataxia with Axonal Neuropathy type 1
TDP1	tyrosyl-DNA phosphodiesterase 1
Δ148 TDP1	truncated TDP1 lacking the first 148 N-terminal amino acid residues
TOP1	topoisomerase 1
TOP1cc	TOP1-DNA covalent complex
TPC	topotecan
