# Supplementary material for: The Influence of TDP1 Inhibitor Usnic Acid Derivative OL9-116 on the Effects of Topotecan in Human Cells
Source: Curr Issues Mol Biol. 2026 Apr 21;48(4):428. doi: 10.3390/cimb48040428 (PMC13114712; doi:10.3390/cimb48040428)
Supplement: Supplementary file 1 [file cimb-48-00428-s001.zip › cimb-4191397-supplementary.pdf]

Supplementary Material

The Influence of TDP1 Inhibitor Usnic Acid Derivative OL9-116 on the Effects of Topotecan in Human Cells

Tatyana E. Kornienko [1], Arina A. Chepanova [1,2], Maria V. Kolobenko [3], Irina A. Chernyshova [1,2], Alexandra L. Zakharenko [1], Artur S. Venzel [2], Nadezhda S. Dyrkheeva [1,3], Andrey V. Markov [1], Rashid O. Anarbaev [1], Konstantin N. Naumenko [1], Olga A. Luzina [4], Nariman F. Salakhutdinov [4], Vladimir A. Ivanisenko [2] and Olga I. Lavrik [1,3,5,*]

1 Institute of Chemical Biology and Fundamental Medicine, Siberian Branch of the Russian Academy of Sciences, 630090 Novosibirsk, Russia; t.kornienko1995@gmail.com (T.E.K.); andmrkv@gmail.com (A.V.M.)

2 Federal Research Centre Institute of Cytology and Genetics, Siberian Branch of the Russian Academy of Sciences, 630090 Novosibirsk, Russia

3 Department of Natural Sciences, Novosibirsk State University, Pirogov St., 2, 630090 Novosibirsk, Russia

4 N. N. Vorozhtsov Novosibirsk Institute of Organic Chemistry of SB RAS, Lavrent'ev ave., 9, 630090 Novosibirsk, Russia; luzina@nioch.nsc.ru (O.A.L.)

5 The Institute of Biology and Biotechnology, Altai State University, Pr. Lenina 61, 656049 Barnaul, Russia

* Correspondence: lavrik@1bio.ru

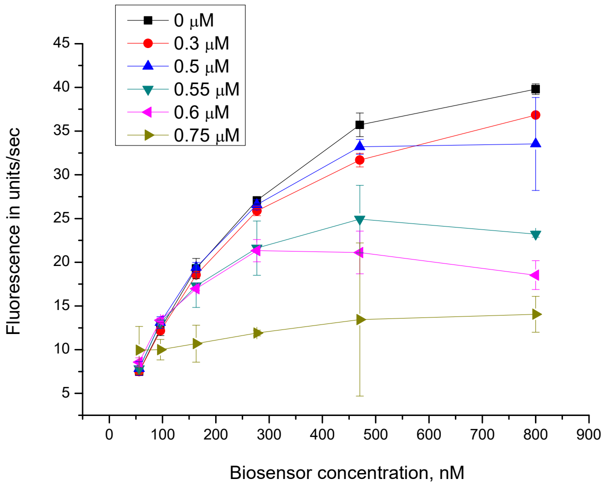

Figure S1. Plots of the reaction rate V versus substrate concentration S at different concentrations of OL9-116

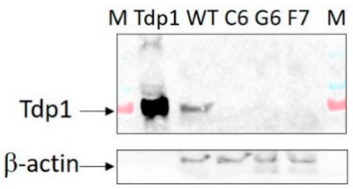

Figure S2. Western-blot analysis of the CRISPR/Cas9-induced deletion of TDP1 in HEK293A cells: whole cell extracts were separated by Laemmli electrophoresis in SDS-PAAG, transferred on nitrocellulose membrane, and probed with rabbit antibody to TDP1 (Thermo Fisher Scientific PA5-27111) or rabbit antibody to β-actin (Abcam 8226-100). Lanes: 1) protein ladder (Thermo Fisher Scientific); 2) purified TDP1 (20 ng); 3) HEK293A WT cells; 4) HEK293A clone C6; 5) HEK293A clone G6; 6) HEK293A clone F7; 7) protein ladder (Thermo Fisher Scientific). In this work, HEK293A clone C6 was used.

Validation of the Molecular Docking Protocol

To validate the molecular docking protocol used in this study, a self-docking benchmark was performed on 14 TDP1–inhibitor co-crystal structures solved by Lountos et al. [1]. These structures (PDB IDs: 6DHU, 6DIE, 6DIH, 6DIM, 6DJD, 6DJE, 6DJF, 6DJG, 6DJH, 6DJI, 6DJJ, 6MJ5, 6N17, 6N19) represent TDP1 in complex with various fragment-like inhibitors bound at the catalytic site. For each structure, the co-crystallized ligand was extracted and re-docked into its protein structure using GNINA with the same parameters as described in Section 2.6. Ten docking poses were generated per structure. The best pose was selected as the one with the highest CNNScore (Table S1, Figure S3).

Experimental dissociation constants (Kd) for nine of the fourteen complexes were obtained from the PDBBind v2020 database [2] and converted to –log Kd values for correlation analysis. Binding affinities were computed as ΔG = ΔGcomplex – (ΔGprotein + ΔGligand) in Rosetta Energy Units (REU) using the ref2015 scoring function. GNINA's minimized affinity (Vina-like empirical score, kcal/mol), CNNaffinity (CNN-predicted pK), and CNNscore (pose quality, 0–1) were also recorded.

Table S1. Re-docking validation results. For each TDP1–inhibitor co-crystal structure, the heavy-atom RMSD (Å) between the best docked pose and the crystallographic ligand coordinates is shown.

**PDB ID**	**RMSD (Å)**	**Best CNNscore**
6DHU	0.319	0.946
6DIE	0.389	0.959
6DIH	0.176	0.964
6DIM	0.128	0.969
6DJD	0.123	0.932
6DJE	0.179	0.949
6DJF	0.350	0.973
6DJG	0.282	0.977
6DJH	0.034	0.906
6DJI	0.348	0.966
6DJJ	0.192	0.975
6MJ5	0.224	0.955
6N17	0.890	0.927
6N19	0.804	0.928

Twelve of the fourteen structures (86%) achieved sub-angstrom RMSD, with a median RMSD of 0.23 Å. The two outliers, 6N17 (0.89 Å) and 6N19 (0.80 Å), correspond to larger naphthoquinone-type inhibitors, where ligand flexibility and extended binding geometry contribute to increased docking difficulty. Notably, even for these outliers, the RMSD values remained below 1 Å, which is well within the commonly accepted threshold for successful docking reproduction. Pearson correlation analysis of the scoring metrics against experimental –log Kd values for nine complexes with available binding data (Table S2) revealed that the AD4 affinity score showed the strongest correlation (r = –0.79), followed by Rosetta interface energy (r = –0.69), supporting the reliability of the AD4 scoring function employed in this protocol. Visual comparison of the best docked poses (cyan) against crystallographic coordinates (gray) for all 14 structures is shown in Figure S3.

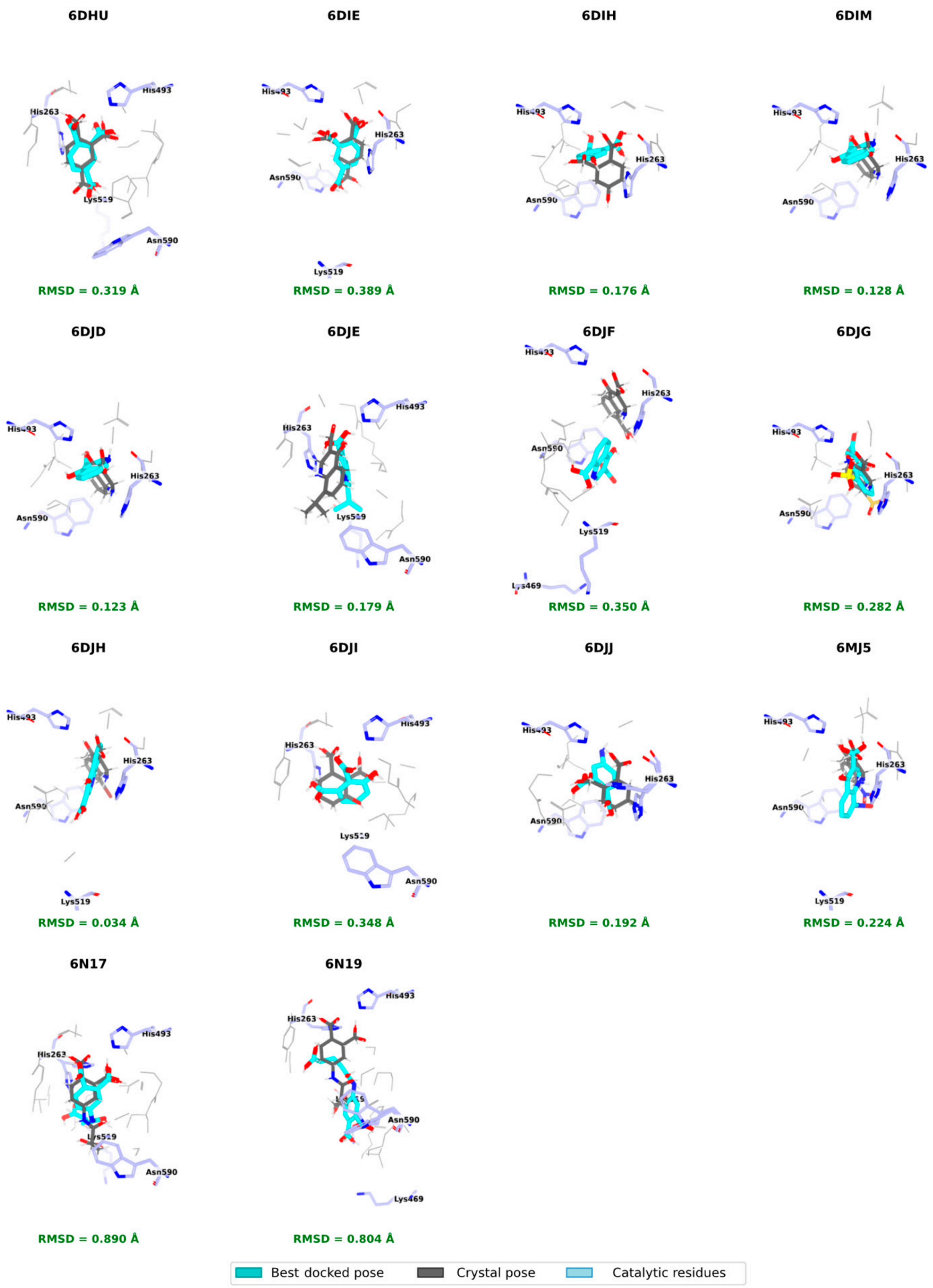

Figure S3. Overlay of GNINA best docked poses (cyan) and crystallographic ligand coordinates (gray) for all 14 TDP1–inhibitor co-crystal structures. Catalytic residues (His263, His493, Lys519, Asn590) are shown as light blue sticks. RMSD values (in Å) are indicated below each panel.

Correlation of Scoring Metrics with Experimental Binding Data

For the nine structures with available experimental –log Kd values from PDBBind, Pearson correlations were computed between pairs of scoring metrics (Table S2).

Table S2. Scoring metrics for the nine TDP1–inhibitor complexes with experimental binding data. ΔG: Rosetta interface energy (REU); Affinity: GNINA minimized affinity (kcal/mol); CNNaffinity: RMSD: best-pose heavy-atom RMSD (Å).

PDB ID	–log Kd	ΔG (REU)	Affinity (kcal/mol)	CNNaffinity	RMSD (Å)
6DHU	4.13	–15.30	–7.03	4.831	0.319
6DIE	4.13	–18.86	–6.82	4.220	0.389
6DIH	3.72	–10.28	–3.92	4.038	0.176
6DIM	3.14	–7.84	–4.40	3.964	0.128
6DJD	3.14	–11.15	–5.09	3.158	0.123
6DJI	3.51	–17.52	–5.60	3.789	0.348
6DJJ	4.10	–25.98	–7.31	3.312	0.192
6N17	4.00	–21.99	–6.96	3.765	0.890
6N19	4.02	–31.33	–6.82	4.385	0.804
Pearson vs -logKd	1	–0.69	–0.79	+0.47	+0.51

The Rosetta interface energy (ΔG) showed a significant Pearson correlation with experimental –log Kd (r = –0.69), indicating that more favorable Rosetta binding energies correspond to higher binding affinities. AD4 affinity score yielded an even stronger correlation (r = –0.79), making it the best single predictor of experimental binding in this benchmark set. GNINA [3, 4] uses the AutoDock4 (AD4) scoring function for conformational sampling and refinement, and applies a CNN scoring function (CNNScore) for final pose ranking. In our benchmark, the AD4 affinity score showed the strongest correlation with experimental binding data (r = −0.79 vs. –log Kd), outperforming both Rosetta interface energy (r = −0.69) and CNNaffinity (r = +0.47), while CNNScore reliably identified geometrically correct poses as reflected by the low re-docking RMSDs (Table S1).

Detailed molecular dynamics protocol

MD simulations were carried out for two catalytic states of TDP1 in complex with a docked inhibitor and a DNA oligonucleotide cofactor (GTT trinucleotide with 3'-phosphate). In the first system, the DNA was covalently bound to His263 NE2; in the second, to Tyr723 OH of a peptide fragment (Lys720–Asp725). The inhibitor was treated as a non-covalent binder.

The AMBER ff14SB force field was used for protein, GAFF 2.11 with AM1-BCC charges for the DNA oligonucleotide and inhibitor, and TIP3P for water. Hydrogen atoms on protein residues were stripped from the input coordinates and rebuilt using the OpenMM Modeller addHydrogens routine (pH 7.0) to eliminate steric clashes present in the initial structure. The covalent protein–DNA phosphoester bond was maintained by a harmonic potential (k = 250,000 kJ/mol/nm^2) with the equilibrium distance taken from the input coordinates; nonbonded exclusions (1-2 and 1-3) were added for atom pairs across the junction, and 1-4 pairs were assigned standard AMBER scaling (electrostatic / 1.2, Lennard-Jones x 0.5) to prevent artificial close-range repulsion. Because the cross-residue bond is invisible to the template-based force field (ignoreExternalBonds), the missing angle and torsion terms spanning the junction were added explicitly: CZ–OH–P (theta_0 = 120 deg, k = 585 kJ/mol/rad^2) and OH–P–O_i (theta_0 = 104 deg, same k) angles, along with CE–CZ–OH–P (periodicity 2, barrier 7.5 kJ/mol) and CZ–OH–P–O_i (periodicity 3, barrier 2.0 kJ/mol) torsions, using parameters derived from GAFF2 os–p5 and AMBER parm99 phosphoester analogues.

In the TOPcc-DNA system, the hydroxyl hydrogen (HH) was fully ghosted to model the bridging ester oxygen: its partial charge was transferred to OH, and all Lennard-Jones, angle, and torsion parameters involving HH were zeroed; the O–H bond constraint was retained for numerical stability. Hydrogen mass repartitioning (4 amu) was applied to enable a 4 fs integration timestep. Each system was solvated in a cubic water box (12 A padding) with 0.15 M NaCl. Energy minimization was performed using the L-BFGS algorithm (tolerance 10 kJ/mol/nm).

Equilibration proceeded in three stages. First, NVT heating from 50 to 300 K was carried out over 100 ps (1 fs timestep) with positional restraints on all solute heavy atoms (k = 10 kcal/mol/A^2). Second, NPT equilibration at 300 K and 1

bar was performed with restraints still applied (2 fs timestep, 50 ps), after which the restraint strength was gradually reduced (0.5x, 0.1x, 0.01x, 0x of the original value, 10 ps each) to allow smooth relaxation of the solute. After a further 50 ps of unrestrained NPT at 2 fs, a brief energy minimization was performed before switching to the production timestep of 4 fs for a final 50 ps NPT stage. Velocities were preserved across timestep transitions to avoid

re-randomization artifacts. Production runs of 100 ns were carried out under NPT conditions (300 K, 1 bar) using the Langevin middle integrator (friction 1 ps^-1) and Monte Carlo barostat. Long-range electrostatics were treated with PME (10 A cutoff). Trajectory frames were saved every 50 ps. For each system, three independent replicas were performed with different initial velocity seeds to assess reproducibility and improve sampling. All simulations were performed in OpenMM 8.1 with CUDA acceleration.

Table S3. Hydrogen bond occupancy (%) between OL9-116 and TDP1 residues during MD simulations. Values represent mean occupancy across three independent 100 ns replicas (R1–R3) ± standard deviation. Only hydrogen bonds present in at least two of three replicas with >1% mean occupancy are shown. Hydrogen bonds were identified using the Baker–Hubbard criteria as implemented in MDTraj.

System	Donor	Acceptor	Mean (%)	±SD (%)	R1 (%)	R2 (%)	R3 (%)
**Phosphohistidine covalent intermediate**	Ser463 OG	OL9-116 O11	29,2	16,4	28,3	13,2	46,0
	Tyr204 OH	OL9-116 O7	14,1	5,7	17,2	17,6	7,4
	Ser463 OG	OL9-116 O13	13,0	6,5	20,6	9,2	9,4
	OL9-116 N2	Tyr204 OH	3,3	2,7	3,0	6,0	0,7
	Trp590 NE1	OL9-116 O11	1,2	1,0	1,8	1,7	0,0
	Trp590 NE1	OL9-116 O13	1,1	1,2	0,9	2,5	0,0
**Pre-catalytic TDP1–TOP1cc complex**	OL9-116 O21	Ser608 O	51,2	6,8	56,0	54,1	43,5
	OL9-116 O21	Ser608 OXT	33,7	0,7	34,0	34,2	32,9
	Asn591 ND2	OL9-116 O17	12,4	13,0	5,0	4,7	27,4
	Asn591 ND2	OL9-116 O25	10,1	9,5	4,2	5,0	21,1
	Ser608 N	OL9-116 O21	2,4	2,4	5,1	0,5	1,7

Supplementary References

[1] Lountos, G.T.; Zhao, X.Z.; Kiselev, E.; Tropea, J.E.; Needle, D.; Pommier, Y.; Burke, T.R.; Waugh, D.S. Identification of a Ligand Binding Hot Spot and Structural Motifs Replicating Aspects of Tyrosyl-DNA Phosphodiesterase I (TDP1) Phosphoryl Recognition by Crystallographic Fragment Cocktail Screening. Nucleic Acids Res. 2019, 47, 10134–10150. https://doi.org/10.1093/nar/gkz515

[2] Wang, R.; Fang, X.; Lu, Y. The PDBbind Database: Collection of Binding Affinities for Protein–Ligand Complexes with Known Three-Dimensional Structures. J. Med. Chem. 2004, 47, 2977–2980. https://doi.org/10.1021/jm030580l

[3] McNutt, A.T.; Francoeur, P.; Aggarwal, R.; Masuda, T.; Meli, R.; Ragoza, M.; Sunseri, J.; Koes, D.R. GNINA 1.0: Molecular Docking with Deep Learning. Journal of Cheminformatics 2021, 13, 43. https://doi.org/10.1186/s13321-021-00522-2

[4] McNutt, A.T.; Li, Y.; Meli, R.; Aggarwal, R.; Koes, D.R. GNINA 1.3: The Next Increment in Molecular Docking with Deep Learning. Journal of Cheminformatics 2025, 17, 28. https://doi.org/10.1186/s13321-025-00973-x

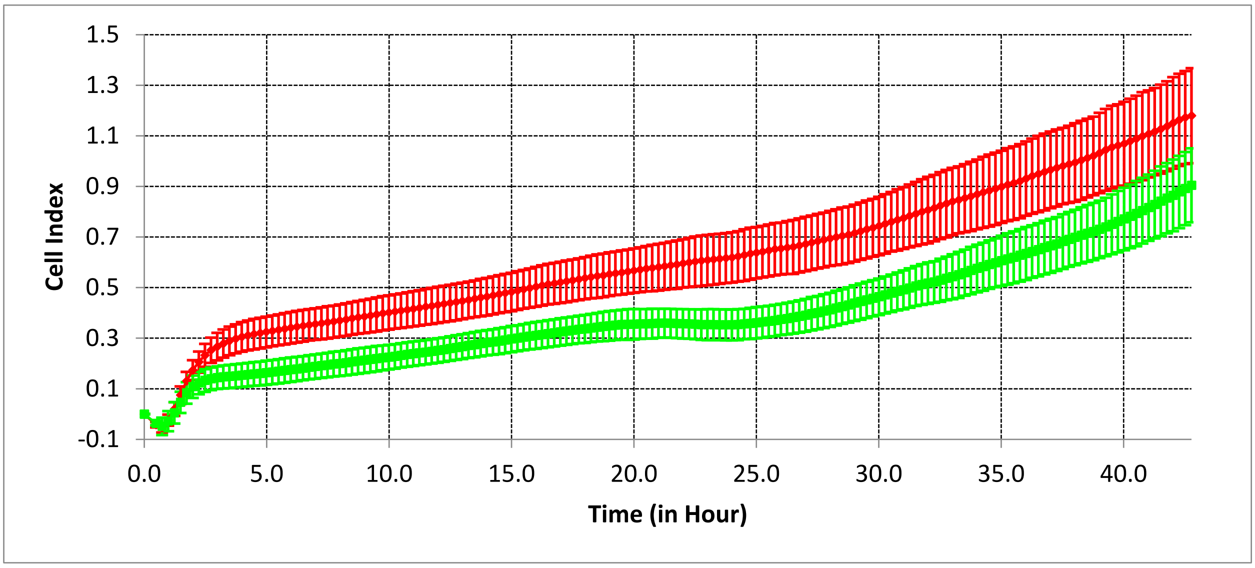

Figure S4. TDP1 knockout does not affect cell doubling time. Red graph - A549 WT cells, green graph - A549 TDP1-KO cells. The doubling time was calculated using the formula DT=T ln2/ln(Xe/Xb)

T is the incubation time (in our case 19 hours, from 23 to 42 hours experiment duration).

Xb is the cell number at the beginning of the incubation time.

Xe is the cell number at the end of the incubation time.
